# Supplementary material for: Detailed molecular and epigenetic characterization of the pig IPEC-J2 and chicken SL-29 cell lines
Source: iScience. 2023 Feb 20;26(3):106252. doi: 10.1016/j.isci.2023.106252 (PMC10018572; doi:10.1016/j.isci.2023.106252)
Supplement: Data S2. Complete homer output for identified motifs in Chicken SL-29, related to Tables 5 and 6 — Homer motif analysis results for histone modifications H3K4me1, H3K4me3, H3K27ac, enhancers, and ATAC-seq of chicken SL-29 cell line. Parameters for possible false positives is as mentioned earlier for S5. [file mmc3.zip › Data_S2/S6/Chicken_SL_29/motif_analyis_enhancer_regions/homerResults/motif5.similar.html]

motif5

## Information for motif5

G
A
C
T
A
C
T
G
T
A
C
G
A
C
G
T
T
C
A
G
T
C
G
A
A
T
C
G
A
C
G
T
T
A
C
G
A
T
G
C
A
T
C
G
A
C
T
G
  
Reverse Opposite:  

T
G
A
C
T
A
G
C
T
A
C
G
A
T
G
C
T
G
C
A
T
A
G
C
A
G
C
T
A
G
T
C
T
G
C
A
A
T
G
C
T
G
A
C
C
T
G
A
  

|  |  |
| --- | --- |
| p-value: | 1e-17 |
| log p-value: | -4.084e+01 |
| Information Content per bp: | 1.671 |
| Number of Target Sequences with motif | 720.0 |
| Percentage of Target Sequences with motif | 24.70% |
| Number of Background Sequences with motif | 8036.3 |
| Percentage of Background Sequences with motif | 18.22% |
| Average Position of motif in Targets | 152.0 +/- 82.6bp |
| Average Position of motif in Background | 150.2 +/- 116.3bp |
| Strand Bias (log2 ratio + to - strand density) | 0.3 |
| Multiplicity (# of sites on avg that occur together) | 1.15 |
| Motif File: | file (matrix) reverse opposite |

### Similar de novo motifs found

|  |  |  |  |  |  |  |  |
| --- | --- | --- | --- | --- | --- | --- | --- |
| Rank | Match Score | Redundant Motif | P-value | log P-value | % of Targets | % of Background | Motif file |
| 1 | 0.951 | G A C T A T C G T C A G C A G T T C A G T C G A A T C G A G C T A T C G A T G C T C A G A T C G A T G C | 1e-17 | -40.257367 | 4.94% | 2.21% | motif file (matrix) |
| 2 | 0.618 | A C G T A C T G C G T A A C T G A C T G A C T G | 1e-16 | -36.972881 | 31.05% | 24.30% | motif file (matrix) |
| 3 | 0.680 | A T C G T A C G A G T C T A G C A G T C T C A G A T G C A G T C C T A G A G T C A G C T A G T C G C T A A G T C A G T C | 1e-10 | -23.664295 | 1.34% | 0.38% | motif file (matrix) |
